# A high-resolution fMRI investigation of BNST and centromedial amygdala activity as a function of affective stimulus predictability, anticipation, and duration

**DOI:** 10.1093/scan/nsz095

**Published:** 2019-12-10

**Authors:** Walker S Pedersen, L Tugan Muftuler, Christine L Larson

**Affiliations:** 1 University of Wisconsin, Milwaukee, WI 53211, USA; 2 Medical College of Wisconsin, Milwaukee, WI 53226, USA

## Abstract

Relative to the centromedial amygdala (CM), the bed nucleus of the stria terminalis (BNST) may exhibit more sustained activation toward threat, sensitivity to unpredictability and activation during anxious anticipation. These factors are often intertwined. For example, greater BNST (*vs* CM) activation during a block of aversive stimuli may reflect either more sustained activation to the stimuli or greater activation due to the anticipation of upcoming stimuli. To further investigate these questions, we had participants (19 females, 9 males) complete a task adapted from a study conducted by Somerville, Whalen and Kelly in 2013, during high-resolution 7-Tesla fMRI BOLD acquisition. We found a larger response to negative *vs* neutral blocks (sustained threat) than to images (transient) in the BNST, but not the CM. However, in an additional analysis, we also found BNST, but not CM, activation to the onset of the anticipation period on negative *vs* neutral trials, possibly contributing to BNST activation across negative blocks. Predictability did not affect CM or BNST activation. These results suggest a BNST role in anxious anticipation and highlight the need for further research clarifying the temporal response characteristics of these regions.

## Introduction

Responding appropriately to differing types of threat is a critical adaptive function of the brain. Threats that are predictable and imminent are likely to trigger an immediate fight or flight response, while threats that are unpredictable and more distant are likely to initiate vigilance behaviors, enabling the organism to further assess the degree and nature of the threat (Fanselow, 1986; Blanchard *et al.,* 1993). The extended amygdala is a set of interconnected subcortical structures, including the centromedial amygdala (CM)—comprised of the central and medial nuclei in the dorsal amygdala—and bed nucleus of the stria terminalis (BNST), thought to play a role in the threat response. Rodent research suggests that the central nucleus of the amygdala (CeA) mediates the rapid response to imminent and predictable threat, while the BNST mediates the response to sustained periods of distant and unpredictable threat (Hitchcock and Davis, 1986, 1991; Fanselow, 1994; Davis *et al.,* 1997; Price, 2005; Resstel *et al.,* 2008; Cai *et al.,* 2012). Based on this research, Davis *et al.* (2010) proposed that the CeA mediates the short-term fear response to imminent threat, while the anxiety response to prolonged or unpredictable threat is initiated via excitatory projections from the CeA to the bed nucleus of the stria terminalis (BNST), which then coordinates the sustained anxiety elicited by this type of threat. Once activated, the BNST inhibits CeA activation, allowing for a transition from a transient to a sustained response to threat.

This model has led to several hypotheses about the functional distinction between the CeA and BNST. One hypothesis is that the CeA exhibits transient activation to briefly presented threat stimuli, while the BNST exhibits sustained activation to prolonged threat (Alvarez *et al.,* 2011; Somerville *et al.,* 2013). Another is that the CeA responds to threat that is temporally predictable, while the BNST responds to threat that is temporally unpredictable (Somerville *et al.,* 2013; Goode and Maren, 2017). Finally, the BNST may be more active during periods of anxious anticipation, while the CeA is more sensitive to the onset of a threatening stimulus (Straube *et al.,* 2007; Somerville *et al.,* 2010, p. 201; Klumpers *et al.,* 2017). However, these questions remain understudied, and further research is needed to determine the stimulus characteristics that elicit CeA and BNST activation, as well as the temporal properties of activation in these areas. Investigating this topic will advance our understanding of psychopathology as altered functioning in these regions is thought to play an important role in anxiety disorders (Rauch *et al.,* 2003; Lebow and Chen, 2016).

Davis *et al.’s* (2010) model of the extended amygdala suggests that the CeA mediates the fear response to signals of imminent danger. According to this conceptualization, the fear response is accompanied by defensive fight or flight behaviors in the face of immediate threat. In contrast, the BNST mediates the anxiety response to periods of potential, sustained or unpredictable threat, including freezing behaviors and sustained vigilance. This model predicts CeA activation toward stimuli that are predictable and brief and BNST activation to stimuli that are unpredictable and sustained, as well as different temporal characteristics in the response patterns in these regions. In this model, the CeA exhibits a response to negative stimuli that has a short onset latency and is brief, while the BNST exhibits more sustained activation, taking longer to initiate and to return to baseline.

In a study that has become influential in the human extended amygdala literature, Somerville *et al.* (2013) investigated amygdala and BNST activation to predictable and unpredictable negative and neutral images. This study employed a mixed block-event-related design in an attempt to tease apart transient and sustained activation. In this task, participants were shown blocks of images that were either negatively valenced or neutral. Temporal predictability was manipulated by having images within blocks separated by numbers that either counted down to the next image onset time or were displayed in random order. This interstimulus interval was jittered, allowing for the data to be analyzed with event-related modeling to investigate transient activation, in addition to modeling activation across blocks to investigate sustained activation. Somerville *et al.* (2013) found clusters in both the left and right amygdala that exhibited transient (i.e. event-related) activation to the onset of individual images, but not sustained activation to blocks of negative images. In contrast, they found a cluster in the right BNST and ventral basal forebrain (VBF) exhibiting greater sustained activation to negative *vs* neutral and unpredictable *vs* predictable blocks of images. This cluster did not exhibit transient activation to individual negative *vs* neutral images. These results were interpreted as supporting the hypothesis that the temporal patterns of amygdala and BNST activation to threat differ.

However, other studies have found event-related BNST activation to threat cues, suggesting that the BNST can exhibit signals of short-latency activation to briefly presented stimuli (Avery *et al.,* 2015; Shackman and Fox, 2016). For example, the BNST is activated during brief threat anticipation periods (Choi *et al.,* 2012; Grupe *et al.,* 2013; Klumpers *et al.,* 2015), as well as briefly presented negatively valenced images (Pedersen *et al.,* 2016; Brinkmann *et al.,* 2018), which carry symbolic signals of threat. On the other hand, the amygdala exhibits sustained activation during blocks of negative stimuli (Sergerie *et al.,* 2008). These findings do not necessarily discount Davis *et al.’s* (2010) model. While this model predicts different temporal characteristics in the patterns of activation between the BNST and dorsal amygdala (which includes the CM), it does not necessarily suggest a sharp double dissociation between these regions (Shackman and Fox, 2016). However, these findings call into question the degree to which the human amygdala and BNST reliably exhibit differential temporal patterns of activation.

Past research suggests that the BNST may exhibit activation during anxious anticipation. Rodent research suggests that the CeA mediates the response to imminent threat (Hitchcock and Davis, 1986, 1991; Fanselow, 1994; Price, 2005), while the BNST mediates the sustained vigilance behaviors to more distant or uncertain threat (Resstel *et al.,* 2008; Davis *et al.,* 2010; Cai *et al.,* 2012). In support of this hypothesis, Klumpers *et al.* (2017) demonstrated BNST activation during anticipation of electric shock, shifting to amygdala activation at the onset of the shock. Straube *et al.* (2007) found that spider phobics exhibit BNST, but not amygdala, activation while anticipating the presentation of images of spiders (*vs* neutral images). Similarly, Somerville *et al.* (2010) found BNST, but not amygdala, activation while participants monitored a graph that indicated the number of future electrical shocks they would receive. However, others have reported amygdala activation during anxious anticipation (Nitschke *et al.,* 2009; Carlson *et al.,* 2011). These findings suggest a need for further research on the roles of the amygdala and BNST in anxious anticipation. These findings also highlight the possibility that BNST activation during blocks of negative stimuli may be driven by anticipation of upcoming stimuli, rather than sustained activation to stimulus onset.

The Davis *et al.* (2010) hypothesis that the CeA responds to imminent threat and the BNST responds to unpredictable, potential threat also suggests that the BNST may exhibit more sensitivity to unpredictable threat than the CeA. Going a step further, Goode and Maren (2017) argue that the BNST is recruited specifically when an aversive stimulus is temporally unpredictable. However, human literature testing whether the BNST and amygdala are differentially recruited by unpredictability is mixed. Alvarez *et al.* (2011) found dorsal amygdala activation to both predictable and unpredictable threat and BNST activation to unpredictable, but not predictable threat. Somerville *et al.* (2013) found that a cluster including the BNST responded to blocks of images with unpredictable *vs* predictable onset times, while amygdala sensitivity to unpredictability depended on individual differences in intolerance of uncertainty. These studies suggest the BNST preferentially responds to unpredictable stimuli, while evidence for an amygdala response to unpredictable *vs* predictable threat is mixed (Hasler *et al.,* 2007; Sarinopoulos *et al.,* 2010). As such, the degree to which the BNST may exhibit more sensitivity to unpredictability than the amygdala remains unclear.

To further investigate these topics, we had participants complete a modified version of the task used by Somerville *et al.* (2013). We first tested Somerville *et al.*’s (2013) findings that the BNST exhibits sustained activation to blocks of negative images, while the amygdala exhibits transient activation to the onset of negative images. We also tested whether the BNST exhibited greater activation to the onset of the anticipation period than the amygdala to explore whether greater BNST activation during blocks of negative stimuli may reflect activation to the anxious anticipation period. To test these questions with greater spatial specificity, we coupled high-resolution 7T imaging (0.98 × 0.98 × 1 mm) with an ROI-based analysis approach. This allowed us to characterize BNST activation with greater accuracy than is possible with standard neuroimaging methods. We chose the CM as our primary amygdala region of interest, because both the CeA and medial nucleus of the amygdala are included in the extended amygdala (Alheid, 2003), because the CM has a similar amount of functional heterogeneity as the BNST (Alheid, 2003; Davis *et al.,* 2010; Fox *et al.,* 2015) and because relative to a CeA ROI, this more inclusive ROI would be more likely to include CeA activation for most participants after accounting for spatial inaccuracies introduced by warping ROIs to each subject’s native space. We also explored activation in the basolateral and superficial amygdala.

**Fig. 1:**
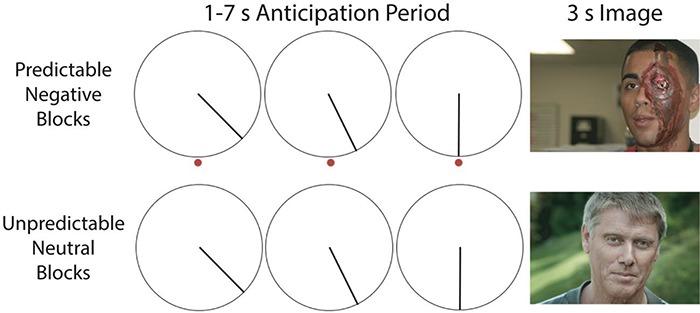
Participants completed a task adapted from Somerville *et al.* (2013). Participants were shown blocks of negatively valenced and neutral images with predictable and unpredictable timings. Predictability was manipulated via the image of a clock, which had a second hand that either progressed toward a specified end-point or simply rotated. Participants completed 13 trials per block, with 2 blocks per run across 4 runs for a total of 2 blocks per condition. Due to restrictions of the IAPS and NAPS user agreements, images displayed in the figure are public domain images, and were not taken from the NAPS or IAPS images used in the study.

Somerville *et al.* (2013) found that the BNST exhibits sustained, but not transient, activation to negative stimuli, while the amygdala exhibits transient, but not sustained, activation. However, other studies demonstrate that the BNST sometimes exhibits a transient response to briefly presented stimuli (Choi *et al.,* 2012; Grupe *et al.,* 2013; Klumpers *et al.,* 2015; Pedersen *et al.,* 2016; Brinkmann *et al.,* 2018) and that the amygdala exhibits a sustained response to blocks of negative stimuli (Sergerie *et al.,* 2008). Given these past studies, we predicted that both the BNST and CM would exhibit both transient and sustained responses to negative stimuli. However, we predicted that in the BNST, signals of sustained activation to blocks of negative stimuli would be greater than signals of transient activation to the presentation of individual images. Conversely, we predicted that in the CM signals of transient activation would be greater than signals of sustained activation. We also predicted that the BNST would exhibit greater activation to negative stimuli presented with unpredictable (*vs* predictable) onset times, while CM activation to negative stimuli would not be affected by predictability.

We also investigated neural activation during the anticipation of negative *vs* neutral images. Based on Davis *et al.’s* (2010) model, and past findings (Straube *et al.,* 2007; Somerville *et al.,* 2010; Klumpers *et al.,* 2017), we expected anticipation of negative *vs* neutral images to elicit BNST activation more strongly than CM activation. We also expected the BNST to exhibit stronger activity during the anticipation of negative images when the timing of image onset was unpredictable *vs* predictable. If the BNST exhibits greater activation during the anticipation period than the CM, this would suggest that the greater activation during blocks of negative images may be partially due to increased activation during anxious anticipation, rather than exhibiting a more general pattern of sustained activation.

## Method

### Participants

Thirty-five undergraduate students at the University of Wisconsin—Milwaukee participated in the study. Three participants withdrew from the study prior to completing the task, two participants were excluded due to fMRI signal loss that affected the BNST or amygdala, one participant was excluded due to excessive motion, and one was excluded due to equipment failure. As a result, data from 28 participants (19 females, 9 males) were included in the analysis. Participants had a mean age of 22.1 years (SD = 5.95). Participants provided consent prior to the study, and all study procedures were approved by the Medical College of Wisconsin institutional review board.

### Task design

Participants completed an fMRI task that was adapted from Somerville *et al.* (2013) (Figure 1). Participants viewed blocks of images taken from the International Affective Picture Set (IAPS; Lang and Bradley, 2007) and the Nencki Affective Picture System (NAPS; Marchewka *et al.,* 2014). Image valence was manipulated with half of the blocks containing negatively valenced images and half containing neutral images. Predictability of image onset was also manipulated with image onset being predictable in half of the blocks. Thirteen images were presented in each block for 3 s each. Blocks were separated by a 20-s fixation period. At the beginning of each block, participants were given a 3-s cue conveying the upcoming block type, for example, the cue would say ‘Unpredictable Negative Block’ before that block type began. Participants were asked to press one of the two buttons to indicate whether the image took place indoors or outdoors. Participants completed 4 runs, with 2 blocks per run, for a total of 2 blocks and 26 images for each condition (predictable negative, predictable neutral, unpredictable negative, unpredictable neutral).

Images used for each condition were pulled from four lists of images. Two of these lists contained negatively valenced images, while two contained neutral images. Images with low valence ratings were selected for negatively valenced images, while images with valence ratings toward the middle of the scale were selected as neutral images. Typical negatively valenced images depicted individuals with severe injuries, surgical procedures or car accidents, while typical neutral images depicted healthy individuals with neutral expressions or objects. In each of these sets, half of the images were taken from the IAPs and half from the NAPS. Additionally, half took place indoors and half outdoors. Sets were also matched for number of images depicting people and number with visible faces. The two negative image sets were matched for valence (NAPS: M = 2.09, SD = 0.35; IAPS: M = 2.04, SD = 0.38) and arousal (NAPS: M = 7.28, SD = 0.41; IAPS: M = 6.33, SD = 0.64) as were the two neutral image sets (for valence NAPS: M = 5.4, SD = 0.57; IAPS: M = 5.42, SD = 0.47 and for arousal NAPS: M = 4.81, SD = 0.47; IAPS: M = 3.51, SD = 0.56). For each participant one negative image set was randomly assigned to each of the negative conditions (predictable and unpredictable), and one neutral image set was assigned to each of the neutral conditions. Order of the conditions was pseudo-randomized, such that the same block was not presented twice in a row.

Images were separated by a 1–7-s (M = 4 s) anticipation period. During this anticipation period, the image of a clock with a rotating second hand was presented. On predictable trials, this second hand rotated toward a red dot, and the image was presented when the hand reached this dot. On unpredictable trials the participants viewed the rotating second hand, but there was no dot to indicate when the image would be presented.

Prior to the task, participants viewed the following set of instructions, along with examples of the clock image:

‘During this task you will see several sets of images. All of the images in each set will be either emotionally neutral or emotionally negative. Before each image a clock will appear to tell you when the next image will be shown. When the second hand reaches the red dot, the next image will be presented. However, during some image series, the clocks will still appear, but they will not have a red dot to tell you when the next image will be shown. On these trials the length of time to the next image will be unpredictable. Before each series of images a screen will appear that will tell you whether the images in the next series will be predictable or unpredictable, and whether the images will be negative or neutral. The only thing you have to do during the task is to decide whether each image takes place indoors or outdoors. For each image: If the image takes place indoors, press 1. If the image takes place outdoors, press 2.’

After each run, participants were asked to report how anxious they were using a nine-point Likert scale for each of the two blocks within that run.

### MRI acquisition

MRI data were acquired on a 7-Tesla MR950 General Electric (GE Healthcare, Waukesha, WI) scanner. High-resolution T1-weighted whole-brain anatomical images were acquired using a BRAVO gradient-echo sequence (inversion time/repetition time/echo time/flip angle/field of view/matrix/slice thickness: 1050 ms/7.972 ms/3.776 ms/5°/220 mm/276 × 276 mm/0.8 mm).

Functional scans were acquired in the coronal plane, with coverage placed to include the regions of interest. A single-shot gradient-echo EPI sequence was used for the functional scans (repetition time/echo time/flip angle/number of excitations/field of view/matrix: 2300 ms/24 ms/73°/1/220 mm/224 × 224; 28 × 1 mm coronal slices; gap: 0 mm; 131 volumes) with voxel resolution of 0.98 × 0.98 × 1 mm. The scan coverage was determined for each participant by positioning the most anterior edge of the coverage just anterior to the amygdala and then checking that coverage spanned at least 5 mm anterior to the anterior commissure to ensure coverage of the BNST. After the fMRI acquisition, an additional single-volume EPI scan with reverse phase encode polarity was collected and used for susceptibility-related distortion correction.

### Anatomical ROIs

**Fig. 2:**
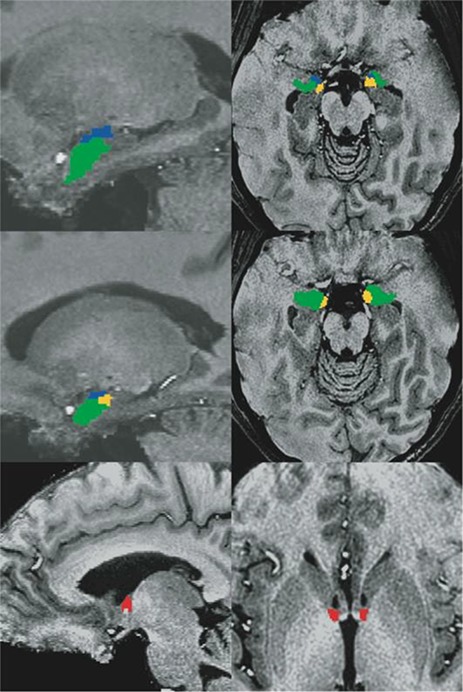
Example of individual BNST (red), CM (blue), superficial amygdala (yellow) and basolateral amygdala (green) ROIs in sagittal (left) and axial (right) views. BNST ROIs were drawn for each subject using anatomical landmarks described by Avery *et al.* (2014). Amygdala ROIs were Amunts *et al.*’s (2005) amygdala subregion maximum probability maps warped into each participant’s native space.

Because our hypotheses concerned activation of the BNST and amygdala, our analysis focused on anatomically defined ROIs for these regions (Figure 2). After N4 bias field correction (Advanced Normalization Tools 2.1; Avants *et al.,* 2009) was applied to the anatomical images, ROIs for the CM, basolateral and superficial amygdala (Amunts *et al.,* 2005) were warped into each participant’s native anatomical space. These ROIs were then visually inspected to ensure accurate alignment. BNST ROIs were traced by hand in AFNI using the anatomical boundaries detailed by Avery *et al.* (2014) (see Figure 2). These anatomical ROIs were down-sampled to the resolution of the EPI images, with EPI voxels being considered part of the ROI if there was more than 50% overlap with the anatomical ROI. The ROIs were then checked by overlaying them on the EPI data and adjusted if necessary, for example, if the ROI encroached onto lateral ventricle. Left BNST ROIs had a mean size of 84 mm^3^, while right BNST ROIs had a mean size of 82.6 mm^3^.

### FMRI analysis

FMRI data were analyzed using AFNI software (Cox, 1996). The first three volumes were discarded to allow for spins to achieve a steady state, and volumes with excessive motion were censored (Euclidean norm >0.3).

Remaining EPI volumes were slice-time corrected and motion corrected. To create a distortion correction template, the third volume from the task EPI data and the third volume of the reverse polarity EPI scan were aligned to each participant’s anatomical scan and warped together using the ‘plus-minus’ option in AFNI’s 3dQwarp. EPI task data were aligned to the anatomical image, non-linearly warped to the distortion correction template, and then to the anatomical image. These three transformations were calculated and applied in a single step to reduce the number of times the data were interpolated. EPI data were converted to percent signal change.

Single-subject BOLD responses at the onset of image presentation were modeled using GLM and a 13.8 s tent function with seven tents. Regressors for the four condition types (predictable neutral, predictable negative, unpredictable neutral, unpredictable negative) were included. Peak activation in response to images was calculated by averaging across tents 3–5. The onset of the anticipation period was also modeled using AFNI’s duration modulation basis function to account for the varying duration of this period. For each subject a second GLM was conducted to model sustained activation during blocks of image presentation for each of the four condition types. This was done using AFNI’s block function—which is a convolution of an incomplete gamma function and a boxcar function—spanning the duration of the blocks. For both models, nuisance regressors were added for low-frequency drift (linear, quadratic and cubic) and motion (L/R, A/P, S/I, roll, pitch, yaw and their derivatives).

### Statistical analysis

Task-evoked anxiety was examined by submitting participants’ self-reported anxiety for each condition type to a 2 × 2 repeated measures ANOVA with valence (negative *vs* neutral) and predictability (predictable *vs* unpredictable) as factors.

Mean BOLD response to the onset of images, the anticipation period, and for activation during blocks of images was extracted for each ROI and condition. These values were entered into SPSS v.24 for further analysis. Values that were greater than 3.5 SD from the mean were counted as outliers and treated as missing. Ten observations (0.3%) met this criterion.

To investigate the degree to which the CM and BNST exhibit transient and sustained activation toward stimulus valence and predictability, these values were submitted to a 2 × 2 × 2 × 2 repeated measures ANOVA with valence (negative *vs* neutral), predictability (predictable *vs* unpredictability), duration (event *vs* block) and area (BNST *vs* CM) as factors. Activation estimates during the anticipation period were also submitted to a 2 × 2 × 2 repeated measures ANOVA with valence (negative *vs* neutral), predictability (predictable *vs* unpredictability) and area (BNST *vs* CM) as factors to investigate the activity in the CM and BNST during anxious anticipation. Similar ANOVAs were also calculated to explore activation in the basolateral and superficial amygdala. These analyses were collapsed across hemisphere, as hemisphere did not interact with any other variable in either of these ANOVAs. Significant interactions were further investigated with follow-up tests, which were Holm–Bonferroni corrected.

To test whether task-evoked anxiety was predicted by activation in the CM and BNST, activation contrast scores were created for the following conditions: negative minus neutral predictable, negative minus neutral unpredictable, unpredictable minus predictable negative and unpredictable minus predictable neutral. Contrast scores for these same conditions were created using self-reported state anxiety scores. For each contrast score, a regression was computed using the neural activation contrast to predict the corresponding contrast for self-reported anxiety. These regressions were Holm–Bonferroni corrected for eight comparisons (four contrasts x 2 areas).

### Voxel-wise analysis

We supplemented our primary ROI-based analysis with a voxel-wise approach. This analysis followed a similar pipeline as the primary analysis, except prior to regression; fMRI data were non-linearly warped to MNI space and subjected to a 2 mm blur (full width at half maximum). Regression was conducted using the same parameters as the primary analysis, and peak activation in response to images was calculated by averaging across tents 3–5. AFNI’s (Cox, 1996) 3dttest++ was used to compute statistics for three contrasts, negative minus neutral blocks, negative minus neutral images and negative minus neutral, at the onset of the anticipation cues. Cluster-based small volume correction for voxels in the amygdala and BNST was calculated. Small volume correction was used because our partial brain imaging was designed to capture only the amygdala and BNST, while any other regions captured were arbitrary. As such, statistics were only computed for voxels lying within these regions. Thresholds for multiple comparison correction were calculated with non-parametric clustering by simulating noise-only *t*-tests with 10 000 permutations using the ClustSim option in 3dttest++. According to these simulations, a cluster size of 16 voxels was necessary to reach a family-wise error rate of *P* < 0.05 while using a voxel-wise alpha of *P* < 0.01.

## Results

### Self-reported anxiety

**Fig. 3:**
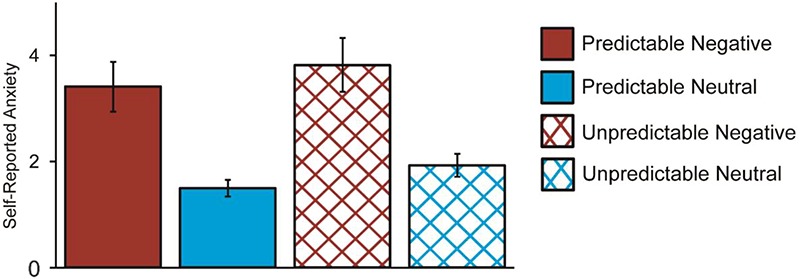
Self-reported anxiety scores for blocks of negative (red) and neutral (blue) images with onset timings that were predictable (solid) or unpredictable (crosshatched). Participants reported significantly more anxiety during blocks of negative *vs* neutral images, *F*(1, 27) = 26.03, *P* < 0.001, *ηp^2^* = 0.491, and during unpredictable *vs* predictable blocks, *F*(1, 27) = 19.35, *P* < 0.001, *ηp^2^* = 0.417. There was no interaction between image valence and predictability, *F*(1, 27) = 0.01, *P* = 0.921, *ηp^2^* < 0.001.

Participants reported more anxiety during blocks of negative than neutral images, *F*(1, 27) = 26.03, *P* < 0.001, *ηp^2^* = 0.491, and more anxiety during blocks with unpredictable than predictable timings, *F*(1, 27) = 19.35, *P* < 0.001, *ηp^2^* = 0.417. There was no interaction between valence and predictability, *F*(1, 27) = 0.01, *P* = 0.921, *ηp^2^* < 0.001. It should be noted, however, that anxiety scores exhibited a floor effect, such that in each condition the modal anxiety score was one (on a Likert scale from one to nine). Means for self-reported anxiety scores are presented in Figure 3.

### Transient and sustained activation in the extended amygdala

**Fig. 4:**
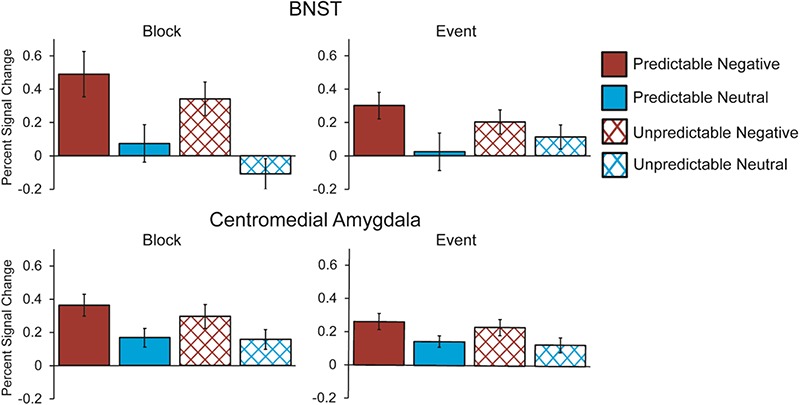
Mean BNST (top) and CM (bottom) BOLD response to negative (red) and neutral (blue) images with predictable (solid) and unpredictable timings (crosshatched) modeled as blocks (left) or as events at image onset (right). While both the BNST and CM exhibited a greater response to negative *vs* neutral images, *F*(1, 26) = 21.261, *P* < 0.001, *ηp^2^* = 0.45, there were no effects involving predictability (*p*s > 0.15). There was a three-way area (BNST *vs* CM) × duration (block *vs* event) × valence (negative *vs* neutral) interaction, which is depicted in Figure 5.

**Fig. 5:**
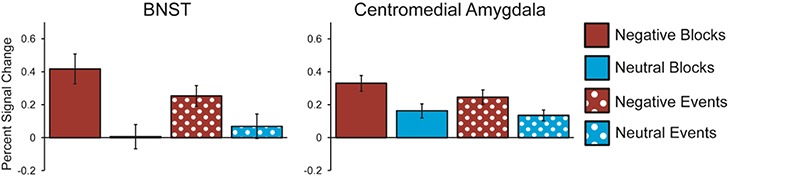
Mean BNST (left) and CM (right) BOLD response to negative (red) and neutral (blue) images modeled as blocks (solid) or as events at image onset (dotted). There was a three-way area (BNST *vs* CM) × duration (block *vs* event) × valence (negative *vs* neutral) interaction, *F*(1, 26) = 7.944, *P* = 0.009, *ηp^2^* = 0.234. The CM exhibited a similar magnitude of response to negative (*vs* neutral) images when data was modeled as blocks and events at image onset, *F*(1, 26) = 1.596, *P* = 0.217, *ηp^2^* = 0.056, suggesting roughly equal signals of sustained and transient activation. However, the BNST exhibited larger response to negative *vs* neutral images when modeled as blocks than when modeled as events, *F*(1, 26) = 16.467, *P* < 0.001, *ηp^2^* = 0.388. This suggests that the BNST exhibits stronger signals of sustained *vs* transient activation to negatively valenced images.

**Fig. 6:**
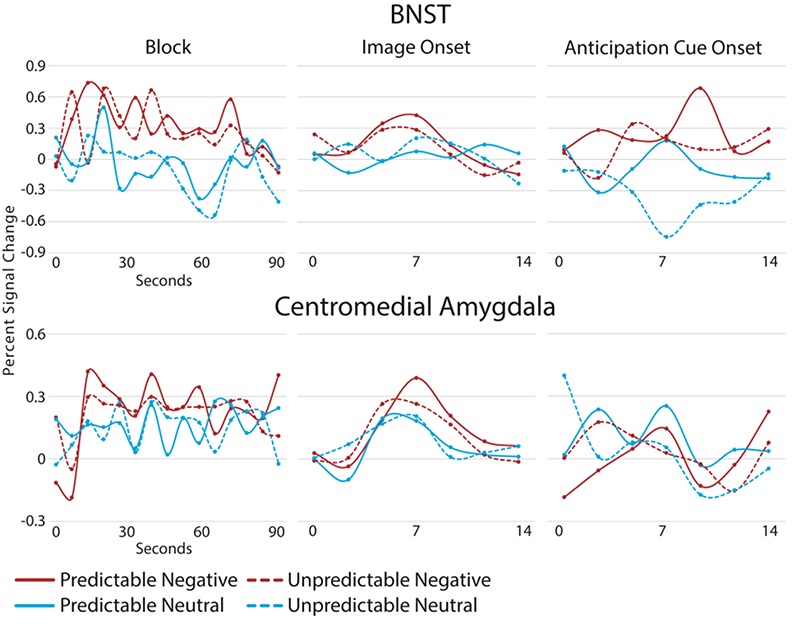
Mean BNST (top) and CM (bottom) BOLD time course during blocks (left), at image presentation (middle) and anticipation cue onset (right) by condition. Time courses were estimated with an AFNI tent function with 15 tent poles for blocks and 7 tent poles for image and anticipation cue onset (tent poles are represented by dots). Only trials with anticipation periods of at least 5 s were used in creating anticipation cue time courses.

A valence (negative *vs* neutral) × predictability (predictable *vs* unpredictability) × duration (event *vs* block) × area (BNST *vs* CM) ANOVA revealed a significant effect of valence, with negative images eliciting more activation than neutral images, *F*(1, 26) = 21.261, *P* < 0.001, *ηp^2^* = 0.45 (Figure 4). There were no significant main effects of duration, *F*(1, 26) = 3.253, *P* = 0.083, *ηp^2^* = 0.111, or area, *F*(1, 26) = 0.047, *P* = 0.83, *ηp^2^* = 0.002. There was an interaction between duration and valence, *F*(1, 26) = 14.733, *P* < 0.001, *ηp^2^* = 0.362. These effects, however, were qualified by a significant area × duration × valence interaction, *F*(1, 26) = 7.944, *P* = 0.009, *ηp^2^* = 0.234. Follow-up ANOVAs demonstrated a significant duration × valence effect in the BNST, *F*(1, 26) = 16.467, *P* < 0.001, *ηp^2^* = 0.388, but not the CM, *F*(1, 27) = 1.596, *P* = 0.217, *ηp^2^* = 0.056. The BNST exhibited a significant response to the valence of both blocks, *t*(26) = 3.812, *P* = 0.002, and individual images (i.e. events), *t*(27) = 2.393, *P* = 0.024. However, the significant area × duration × valence interaction demonstrates that the difference between the valence effects for blocks and events was greater in the BNST than in the CM. This suggests that while the BNST exhibits both transient and sustained signals of activation toward negative valence, the strength of the sustained signal is larger than that of the transient signal. On the other hand, in the CM signals of transient and sustained activation toward negative valence are roughly equal in strength (see Figure 5). There were no significant effects involving predictability (*p*s > 0.15), and all other effects in this ANOVA were non-significant (*p*s > 0.19). Figure 6 depicts the time course of the BNST and CM response during blocks and at image onset.

### Transient and sustained activation in superficial and basolateral amygdala

Task activation in the superficial amygdala was investigated by a valence (negative *vs* neutral) × predictability (predictable *vs* unpredictability) × duration (event *vs* block) ANOVA. There were no main effects of valence, *F*(1, 27) = 3.182, *P* = 0.086, *ηp^2^* = 0.105; predictability, *F*(1, 27) = 0.026, *P* = 0.874, *ηp^2^* = 0.001; or duration, *F*(1, 27) = 0.51, *P* = 0.481, *ηp^2^* = 0.019. There was a significant predictability × valence interaction, *F*(1, 27) = 6.742, *P* = 0.015, *ηp^2^* = 0.2, with a significant effect of valence in the predictable, *t*(27) = 3.034, *P* = 0.011, but not unpredictable condition, *t*(27) = −0.437, *P* = 0.665. There was also a trend toward a duration × valence interaction, *F*(1, 27) = 3.746, *P* = 0.063, *ηp^2^* = 0.122, with a trend toward a response for negative *vs* neutral blocks, *t*(27) = 2.127, *P* = 0.085, but not for the onset of negative *vs* neutral images, *t*(27) = 0.475, *P* = 0.639. There were no other significant effects in this ANOVA (*P* > 0.4).

We also examined activation in the basolateral amygdala with a valence (negative *vs* neutral) × predictability (predictable *vs* unpredictability) × duration (event *vs* block) ANOVA. The basolateral amygdala exhibited a greater response for the negative *vs* neutral condition, *F*(1, 25) = 9.042, *P* = 0.006, *ηp^2^* = 0.266. There were no main effects of duration, *F*(1, 25) = 0.48, *P* = 0.495, *ηp^2^* = 0.019, or predictability, *F*(1, 25) = 0.266, *P* = 0.61, *ηp^2^* = 0.011. There were also no significant interactions (*P* > 0.22).

### Extended amygdala activation during anticipation period

**Fig. 7:**
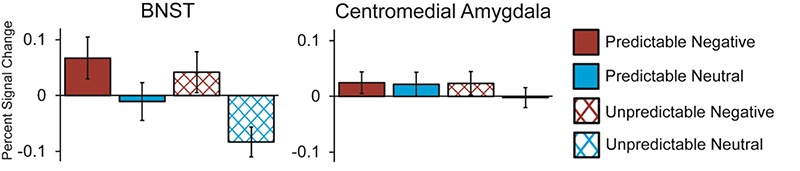
Mean BOLD response in BNST and CM to onset of anticipation period as participants awaited the onset of negative (red) and neutral (blue) images. The BNST exhibited activation during anticipation of negative *vs* neutral images, *t*(27) = 3.87, *P* = 0.001, while the CM did not, *t*(27) = 0.847, *P* = 0.404. Activation during the anticipation period was not affected by the temporal predictability of stimulus onset in either the BNST or CM.

A valence (negative *vs* neutral) × predictability (predictable *vs* unpredictability) × area (BNST *vs* CM) ANOVA for activation during the anticipation period revealed a main effect of valence, *F*(1, 27) = 12.51, *P* = 0.001, *ηp^2^* = 0.317. However, this effect was qualified by a significant area × valence interaction, *F*(1, 27) = 8.804, *P* = 0.006, *ηp^2^* = 0.246. Follow-up tests revealed BNST activation during the anticipation of negative (*vs* neutral) images, *t*(27) = 3.87, *P* = 0.001, but not CM activation, *t*(27) = 0.847, *P* = 0.404 (see Figure 7). There was no significant main effect of area, *F*(1, 27) = 0.834, *P* = 0.369, *ηp^2^* = 0.03, or predictability, *F*(1, 27) = 2.243, *P* = 0.146, *ηp^2^* = 0.077, and no interactions involving predictability, (*p*s > 0.37).

### Activation during anticipation period in superficial and basolateral amygdala

A valence (negative *vs* neutral) × predictability (predictable *vs* unpredictability) ANOVA was used to investigate superficial amygdala activation during the anticipation period. There were no main effects of valence, *F*(1, 27) = 3.24, *P* = 0.083, *ηp^2^* = 0.107, or predictability, *F*(1, 27) = 0.244, *P* = 0.625, *ηp^2^* = 0.009, and no valence × predictability interaction, *F*(1, 27) = 0.048, *P* = 0.829, *ηp^2^* = 0.002.

Basolateral amygdala activation during the anticipation period was also investigated with a valence (negative *vs* neutral) × predictability (predictable *vs* unpredictability) × duration (event *vs* block) ANOVA. There were no main effects of valence, *F*(1, 25) = 0.653, *P* = 0.427, *ηp^2^* = 0.025, or predictability, *F*(1, 25) = 0.987, *P* = 0.33, *ηp^2^* = 0.038, and no valence × predictability interaction, *F*(1, 25) = 0.006, *P* = 0.94, *ηp^2^* < 0.001.

### Neural activation and task-evoked anxiety

Regressions were run to examine whether changes in self-reported anxiety across conditions predicted changes in CM and BNST block activation for the following contrasts: negative minus neutral predictable, negative minus neutral unpredictable, unpredictable minus predictable negative and unpredictable minus predictable neutral. Changes in anxiety across conditions were not predicted by changes in neural activation in the BNST (*p*s = 1). Surprisingly, there was a trend toward a negative association between self-reported anxiety and CM activity during unpredictable minus predictable negative blocks, *β* = −0.486, *t*(26) = −2.832, *P* = 0.072. This suggests that individuals reporting a greater change in anxiety for unpredictable *vs* predictable negative blocks exhibited a reduced change in CM activity across these conditions. CM activity was not related to changes in self-reported anxiety for any other contrast (*p*s = 1).

### Voxel-wise analysis of transient and sustained activation to valence

**Fig. 8:**
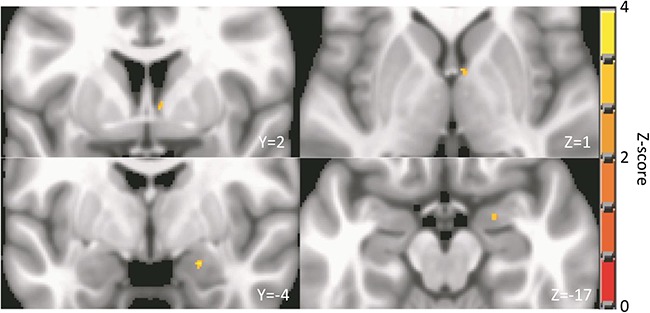
A negative minus neutral block contrast revealed a cluster of activation in the right BNST (top), while a negative minus neutral event contrast yielded a cluster in the right amygdala (bottom), after cluster-based correction for multiple comparison (voxel-wise *P* < 0.01, family-wise *α* = 0.05, cluster size ≥16 voxels). This analysis was restricted to voxels within the amygdala and BNST. Reported slice coordinates are in MNI LPI space.

We supplemented our ROI analysis with a voxel-wise approach. In this analysis, we investigated transient and sustained activation to valence in the amygdala and BNST with two voxel-wise *t*-tests: negative minus neutral blocks and negative minus neutral events (i.e. image onset). The negative minus neutral block contrast revealed a single cluster of positive activation in the dorsal BNST (Figure 8; MNI LPI coordinates for peak: 6, 2, 1).

The negative minus neutral event contrast yielded a single cluster of positive activation, in the amygdala (Figure 8; MNI LPI coordinates for peak: 22, −3, −19). Overlaying this cluster on the Tyszka and Pauli (2016) atlas of the amygdala nuclei revealed that it lies at the intersection of the basolateral nuclei, the anterior amygdaloid area and corticomedial nuclei.

We also tested the activation during the anticipation period with a negative minus neutral contrast for the onset of the anticipation cues. There were no significant clusters of activation for this contrast.

## Discussion

Based on past studies, we predicted that in the BNST signals of sustained activation to blocks of negative stimuli would be greater than signals of transient activation to individual images, while in the CM signals of transient activation would be greater than signals of sustained activation. However, we found that in the CM there was no difference in the magnitude of the transient response to the onset of negative *vs* neutral individual images and the sustained response to blocks of negative images. In contrast, while the BNST also exhibited both transient and sustained activation, the BNST sustained response to blocks of negative images was significantly larger than the transient response to individual images. In addition, we found no significant interaction between valence and duration in the basolateral or superficial amygdala, although there was a trend toward a larger valence effect for blocks than images in the superficial amygdala.

These findings differ from those of Somerville *et al.* (2013) which imply a double dissociation, with the amygdala exhibiting transient, but not sustained signals of activation, and the BNST exhibiting sustained but not transient activation to negative *vs* neutral images. However, these findings do align well with the overall literature which demonstrate that the BNST exhibits signals of transient activation to briefly presented negative stimuli (Choi *et al.,* 2012; Grupe *et al.,* 2013; Avery *et al.,* 2015; Klumpers *et al.,* 2015; Pedersen *et al.,* 2016; Shackman and Fox, 2016; Brinkmann *et al.,* 2018) and that the amygdala exhibits signals of sustained activation to blocks of negative stimuli (Sergerie *et al.,* 2008). These findings also align with points made by Shackman and Fox (2016), who argue that the Davis *et al.* (2010) model should not be ‘recast as a simple double-dissociation’ (p. 8051).

We also found that the BNST exhibited activity during the anticipation of upcoming negative *vs* neutral images, while the CM did not. We found no evidence of activation to valence during the anticipation period in the basolateral or superficial amygdala. These findings support studies demonstrating activation in the BNST during periods of anxious anticipation (Straube *et al.,* 2007; Somerville *et al.,* 2010; Klumpers *et al.,* 2017). This is also in line with Davis *et al.*’s (2010) model, which states that the CM mediates the response to imminent threat, while the BNST mediates sustained vigilance toward threats that are more temporally or spatially distant.

Given that the BNST exhibited greater activation to the onset of anticipation cues, differences in BNST and CM activation patterns for negative blocks and events may be driven by different response patterns to the anticipation period, rather than different temporal response characteristics of the CM and BNST. If the BNST exhibits activation to negative *vs* neutral anticipation periods, this activation may contribute to the finding of BNST activation across blocks. Thus, our finding that the BNST exhibited greater activation to valence across blocks may not necessarily suggest that the BNST intrinsically exhibits more sustained activation but may stem from a combination of transient activation to the onset of the anxious anticipation period and transient activation to the negative images. Future research is needed to examine whether the CM and BNST exhibit different temporal patterns during the presentation of aversive stimuli, as well as during anxious anticipation, as independent research questions.

Davis *et al.*’s (2010) model suggests that the BNST may be more sensitive to unpredictable negative stimuli than the CM, and Goode and Maren (2017) view temporal unpredictability as the primary feature of an aversive stimulus that determines BNST recruitment. We predicted that the CM would respond to negative images regardless of predictability, while the BNST would exhibit a greater response to unpredictable than predictable negative images. However, we found that predictability did not affect activity in either the BNST or the CM and did not interact with any other variables, although there was an effect of valence for the predictable, but not the unpredictable condition in the superficial amygdala. These results differ from those of Alvarez *et al.* (2011) who found that the BNST responded to unpredictable, but not predictable threat, while the amygdala response to threat was not affected by predictability. While Alvarez *et al.* (2011) investigated the neural response to threat of shock, our study investigated the neural response to negatively valenced images. As such, one possible explanation for the apparent differences is that the predictability of a physical threat (i.e. shock) may be more salient than the predictability of an aversive image, which contain signals of threat, but do not represent a threat themselves. This, however, would not account for Somerville *et al.*’s (2013) finding that a cluster including the BNST responded to the predictability of the onset of images, regardless of image valence. One important consideration is that Somerville *et al.* (2013) used a whole-brain approach and reported findings from a cluster that included the BNST and other regions of basal forebrain. In contrast, the current study employed high-resolution imaging and an ROI approach to localize BNST function with greater spatial specificity. Future research employing high-resolution imaging is needed to further clarify the circumstances under which predictability affects BNST activation.

One limitation to our task design is that it employs negative images, rather than a more salient threat stimulus, such an electric shock. The amygdala and BNST reliably respond to negative images (Pedersen *et al.,* 2016; Pedersen *et al.,* 2017), and this response likely subserves the role of these regions in detecting and responding to threat. However, the amygdala and BNST may exhibit different responses to negative images, which carry symbolic representations of threat, than to stimuli that represent a more proximal threat. For example, the temporal predictability of a physical threat may be more salient than the predictability of a negative image.

Another limitation of our study design is that there was no interstimulus interval between the anticipation cues and image presentation. Our design efficiency testing suggested that the variable length of the anticipation cues was enough to independently estimate activation to the onset of the cues and the onset of images. However, if images elicited sustained activation, activation from previous images carried over into the anticipation period is possible, affecting estimates of activation to the anticipation cues. Future studies should investigate BNST activation during periods of anxious anticipation with fixation periods separating anticipation periods from the presentation of the prior stimulus.

Our results partially replicate those of Somerville *et al.* (2013) by showing that the BNST exhibits larger signals of sustained *vs* transient activation, while signals of sustained and transient activation are of similar magnitude in the CM. This result could be seen as bridging the gap between studies showing that the BNST exhibits greater signals of sustained *vs* transient activation (Alvarez *et al.,* 2011; Somerville *et al.,* 2013) and those demonstrating an event-related response to briefly presented stimuli in the human BNST (Choi *et al.,* 2012; Grupe *et al.,* 2013; Klumpers *et al.,* 2015; Pedersen *et al.,* 2016; Brinkmann *et al.,* 2018). However, our finding that the BNST exhibited greater activation to the onset of the anxious anticipation period suggests that the observed activation to blocks of negative *vs* neutral images may be due to a combination of transient activation to negative *vs* neutral images and to the onset of the negative *vs* neutral anticipation periods. Thus, while supporting studies demonstrating a BNST role in anxious anticipation (Straube *et al.,* 2007; Somerville *et al.,* 2010; Klumpers *et al.,* 2017), our findings also highlight a need for further research disentangling the temporal response characteristics of the amygdala and BNST from differential activation during periods of anxious anticipation. We also found no evidence that the amygdala or BNST response to negatively valenced stimuli is affected by the temporal predictability of stimulus onset. This highlights the need to further investigate the circumstances under which predictability affects the response to valence in these regions. Overall, our results support the role of the BNST (*vs* CM) in the anticipation of threat but highlight the need for additional examination of the temporal response characteristics of these regions and of the engagement of these structures in response to unpredictable threat. Further clarifying the roles of the amygdala and BNST in the human threat response is necessary for better understanding the neural mechanisms underlying mood and anxiety disorders.
